# Hyperglycaemia in pregnancy and offspring blood pressure: a systematic review and meta-analysis

**DOI:** 10.1186/s13098-023-00978-2

**Published:** 2023-01-19

**Authors:** Xinyue Zhang, Yinlin Wang, Wenhan Xiao, Denan Jiang, Jiali Zhou, Xinxin Ye, Shiqi Wu, Shuting Li, Peige Song

**Affiliations:** 1 School of Public Health and Women’s Hospital, Zhejiang University School of Medicine, Zhejiang University, Hangzhou, 310058 Zhejiang China; 2 Stomatology Hospital, School of Stomatology, Zhejiang University School of Medicine, Zhejiang Provincial Clinical Research Center for Oral Diseases, Key Laboratory of Oral Biomedical Research of Zhejiang Province, Cancer Center of Zhejiang University, Hangzhou, China; 3 International School of Medicine, Zhejiang University, Yiwu, Zhejiang China; 4 Department of Sport and Exercise Science, College of Education, Zhejiang University, Hangzhou, Zhejiang China; 5 Department of Epidemiology, Mailman School of Public Health, Columbia University, New York, NY USA

**Keywords:** Blood pressure, Gestational diabetes mellitus, Offspring of diabetic pregnancy, Systematic review, Meta-analysis

## Abstract

**Background:**

Hyperglycemia in pregnancy (HIP) is suggested to be a risk factor for elevated blood pressure (BP) in offspring. However, the empirical evidence was mixed. Thus, this systematic review and meta-analysis was conducted to synthesize current evidence assessing the association between HIP and BP in offspring.

**Methods:**

We searched PubMed, MEDLINE, and Embase to identify articles published from inception until 9 February 2021. A random-effects meta-analysis was performed to calculate a pooled effect size and 95% confidence interval (CI). Furthermore, the effects were evaluated separately while grouping by the offspring’s sex, region, economic level, published year, insulin treatment status, and BP measurement. Each article was independently reviewed for quality.

**Results:**

Of 3385 citations identified, 23 studies involving 88695 offspring were included. The study found that the offspring of women with HIP had an increased level of both systolic blood pressure (SBP; mean difference 1.90, 95% CI 1.09 to 2.70 mmHg, P < 0.001) and diastolic blood pressure (DBP; mean difference 0.87 mmHg, 95% CI 0.11 to 1.17 mmHg, P = 0.02) compared with those whose mothers with normal blood glucose during pregnancy. According to subgroup analyses, gestational diabetes mellitus (GDM) appeared to have varied impacts on offspring BP by sex of offspring, region and economic level of family, published year, maternal insulin treatment status, and BP measurement.

**Conclusion:**

Current evidence showed that HIP was associated with an elevated BP in offspring. Prenatal interventions targated on reducing HIP might be beneficial for controlling for offspring BP.

**Supplementary Information:**

The online version contains supplementary material available at 10.1186/s13098-023-00978-2.

## Introduction

Hyperglycemia in pregnancy (HIP) is a kind of hyperglycemia first detected at any time during pregnancy. It can be categorized into two subtypes: one is diabetes mellitus in pregnancy (DIP), including type 1 diabetes mellitus (T1DM) and type 2 diabetes mellitus (T2DM); another is gestational diabetes mellitus (GDM), encompassing pre-existing and developed diabetes during pregnancy [1, 2]. HIP is a prevalent medical complication during gestation [3]. According to the survey conducted by the International Diabetes Federation, over 21.1 million (16.7%) live births to women had HIP worldwide in 2021, with 80.3% of GDM among identified HIP [4]. Meanwhile, the prevalence of GDM varies worldwide, ranging from 6.6% in Japan and Nepal to 45.3% in the United Arab Emirates, and it is expected to rise in most countries [5–10].

A growing body of epidemiologic evidence suggested that HIP was associated with a cluster of long- and short-term adverse maternal outcomes, including preeclampsia, gestational hypertension, and T2DM [11, 12]. Apart from its direct risks to mothers, HIP has also been found to be associated with adverse fetal and neonatal outcomes, such as neonatal metabolic disturbances, fetal macrosomia, stillbirth, and other complications [13, 14]. Studies have shown that the absolute risk of these short-term neonatal consequences in family with GDM mothers ranged from 1.8% for shoulder dystocia to 16.6% for neonatal adiposity [15]. Although HIP may disappear after pregnancy [16], its harm to the next generation could be long-lasting. For instance, a population-based study with 40 years of follow-up found that the offspring of diabetic mothers had a 29% higher rate of early-onset cardiovascular disease [17]. A longitudinal study revealed positive associations between maternal glucose levels and child adiposity [18].

Abundant research indicates that women with HIP provide a fetal environment that may enhance offspring susceptibility to various chronic diseases and contribute to the progression of complex chronic diseases in offspring[19, 20]. Among these diseases, high blood pressure (BP), defined as an increase in arterial systolic and/or diastolic blood pressure at rest, is one of the major chronic diseases threatening human health. The positive association between maternal GDM and offspring BP has been repetitively indicated in recent years and has attracted increasing attention from researchers [6, 17, 18, 21]. Such association has been confirmed by a meta-analysis published in 2012. Aceti, et al. This study demonstrated the association between maternal diabetes and offspring systolic BP from thirteen cohort studies by comparing BP of offspring born to diabetic mothers with that of controls, independent of the obesity [22].

Despite the previously established evidence, it is still necessary to update the original article considering a surge in literature after 2012 [22]. In addition, neither the methodological quality nor the clinical outcomes have been systematically and thoroughly summarized, and the overall evidence remains inconclusive. Therefore, the correlation between HIP and offspring BP should be further synthesized based on an updated analysis. A systematic review and meta-analysis was conducted in the present study to assess the potential association of maternal hyperglycemia and BP in the offspring. If possible, the potential factors moderating the correlation between HIP and offspring BP were also explored.

## Methods

This systematic review and meta-analysis was undertaken following the Preferred Reporting Items for Systematic Reviews and Meta-Analyses (PRISMA) statement [23] and Meta-analyses Of Observational Studies in Epidemiology (MOOSE) guidelines [24]. The review protocol was preregistered in PROSPERO (CRD 42021236328).

### Search strategy and study selection

Two researchers (XZ and YW) independently conducted a literature search in PubMed, MEDLINE, and Embase by utilizing search terms including gestational diabetes (gestational diabetes or GDM or pregnancy glycemic index or Pregnancy-Induced Diabetes), blood pressure (blood pressure or hypertension), and children (children or adolescents or offspring) from inception until 9 February 2021. In addition, reference lists of included papers and related systematic reviews were further gone through to identify eligible sources. No language or geographic restrictions were applied. It was limited to human studies that excluded twins. The detailed search strategies are listed in Text S1, Additional file 1.

The inclusion criteria were: (i) cohort study design; (ii) studies that explored the effects of HIP on offspring SBP and DBP. The exclusion criteria were: (i) papers without full text; (ii) animal studies, randomized-controlled trials, cross-sectional studies, non-in vitro studies, or non-original studies (i.e., reviews, case reports, and protocols); (iii) studies with incomplete or insufficient data; (iv) multiple publications of the same research. When the same cohort was recruited in several studies, the one with the most comprehensive results or the largest sample size was consistently selected.

After removing duplicates from different electronic databases, two researchers (XZ and YW) independently screened the titles and abstracts of retrieved records, followed by a full-text review. Researchers resolved disagreements through discussion until they reached a consensus.

### Data extraction

The following data were extracted from each of the included studies: (i) Study characteristics: first author, published year, study setting, and study design; (ii) Maternal diabetes type, definition, screening method, and treatment; (iii) Child BP measurement and the outcomes of offspring, which were classified by study location, World Health Organization (WHO) region [25], World Bank (WB) income region [26], sex, BP measurement, and insulin treatment. It is worth mentioning that the data from some retrieved studies were derived from a previous review [22]. Two reviewers (XZ, YW) independently extracted data from the included articles. Another reviewer (XY) further discussed the discrepancies until a consensus was achieved.

### Quality assessment

NIH Quality Assessment Tool for Observational Cohort and Cross-sectional Studies (National Heart, Lung, and Blood Institute) was utilized to evaluate the methodological quality of the included studies [6]. The assessment is based on 14 criteria focusing on the key concepts of internal validity, with a total score of 14 points. Based on the overall quality points, included studies were categorized into low- (≤ five points), medium- (six to nine points), and high-quality groups (≥ ten points). Researchers resolved disagreements through discussion.

### Data synthesis and analysis

Two researchers (YZ and YW) analyzed the association between HIP and offspring BP using Review Manager (RevMan) 5.3. Specifically, HIP was identified by WHO standardized diagnostic criteria [27], that is, classified as following (i) or (ii): (i) DIP: an elevated fasting plasma glucose (≥ 7.0 mmol/l), or a 2-h plasma glucose ≥ 11.1 mmol/l following a 75 g oral glucose load, or a random plasma glucose ≥ 11.1 mmol/l in the presence of diabetes symptoms; (ii) GDM: fasting plasma glucose 5.1–6.9 mmol/l, a 1 h plasma glucose ≥ 10.0 mmol/l following a 75 g oral glucose load, or a 2 h plasma glucose 8.5–11.0 mmol/l following a 75 g oral glucose load. The SBP and DBP were analyzed separately to assess the offspring’s BP levels. Since the outcome indexes (SBP and DBP) of this study were continuous variables, the mean difference (MD) between the offspring of diabetic mothers versus their controls and 95% confidence interval (CI) of MD were used to estimate the association.

The heterogeneity among studies was evaluated using Cochran’s Q test and the I^2^ statistic, defining a statistically significant heterogeneity as P-value < 0.05 or I^2^ ≥ 50%. A fixed-effect model was performed on the condition that no statistically significant heterogeneity was presented; otherwise, the random-effects model was utilized to offer more conservative estimations. Thus, all studies were analyzed with a random-effects model in this study to determine the estimated effect [28]. The pooled difference was weighed by the inverse of its variance to take the cross-studies variance into account [29].

Meanwhile, meta-regression and subgroup analyses were performed to analyze potential sources of heterogeneity. Subgroup analyses were carried out based on sex, WHO region, the WB income region, published year, insulin treatment, and BP measurement. To investigate a proximate time trend, the studies were divided roughly and equally into two groups based on the number of publications, using 2010 as the cut-off year. BP measurement data were divided according to manual or digital methods. A funnel plot was generated, of which symmetry suggested no evidence of publication bias. To evaluate the stability of the main analysis, a sensitivity analysis was carried out by sequential removal of each study from the analysis. It is deemed that statistical significance at a P-value < 0.05, and all P-values were two-tailed. The pooled results were all presented in the forest plot where the significant difference exists when the 95% CI of studies overlap with the y-axis [30].

## Result

### Study selection

**Fig. 1:**
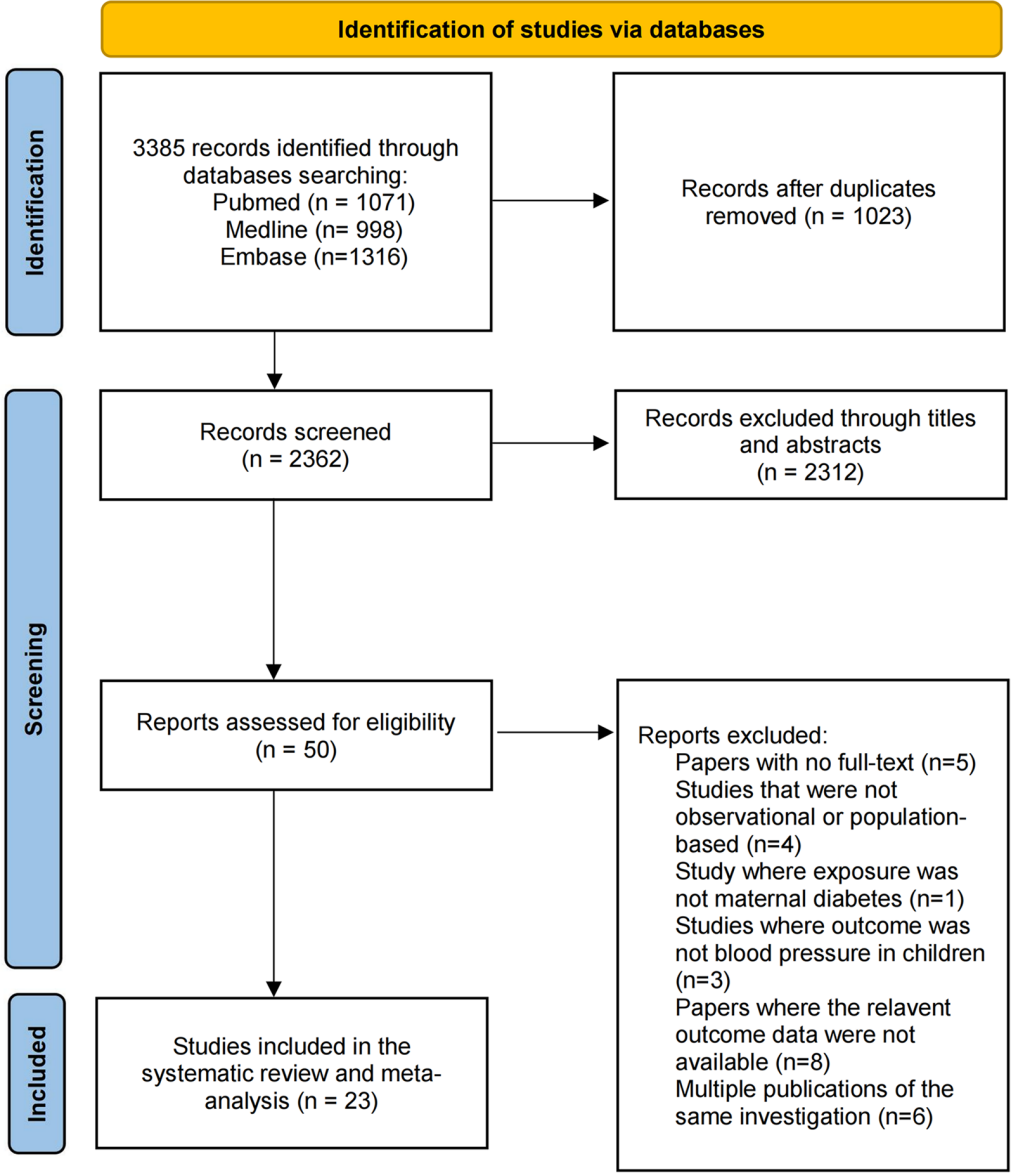
PRISMA flow diagram of screening and selection process

As outlined in Fig. 1, our initial literature search identified a total of 3385 records. After the removal of duplicates, 2362 records were screened by title and abstract, leaving 50 potentially eligible records for full-text review. Finally, the search yielded 23 studies for data extraction (Fig. 1 and Table S1 in Additional file 1).

### Study characteristics

All included studies in this systematic review and meta-analysis were cohort studies [18, 21, 31–51], of which 20 [18, 21, 32–47, 49, 51] were described as prospective cohorts and 3 [31, 48, 50] were retrospective studies. Complete data on offspring SBP were available from 20 studies [18, 21, 31–39, 41–44, 46–48, 50, 51] and on DBP from 18 studies [18, 21, 31–39, 41, 42, 44, 46–48, 50]. In addition, one of the included studies [36] had two cohorts and the other one [42] included three. Apart from the data on GDM and offspring BP, authors provided separate data for T1DM in five original studies [39, 41, 44, 46, 50] and T2DM in one study [32], respectively. The regions included were the European Region (EUR; n = 9, 39.1%), Eastern Mediterranean Region (EMR; n = 1, 4.3%), Region of the Americas (AMR; n = 7, 30.4%), South-East Asia Region (SEAR; n = 3, 13.0%) and Western Pacific Region (WPR; n = 3, 13.0%). 17 studies were conducted on high-income countries (HICs) which accounted for the majority of participants (73.9%). Three (13.0%) studies [18, 33, 36] were conducted in upper-middle-income countries (UMICs), and the remaining three (7.4%) studies [32, 37, 38] were in lower-middle-income countries (LMICs). A description of all studies’ characteristics was provided in Table 1.

**Table 1:** Studies included in the systematic review investigating the association between exposure to HIP and offspring BP

Study, year	Study setting	Study design	Study details	Maternal diabetes type	Diabetes definition	Diabetes treatment	**BP measurement**	**Outcome**	**Number of participants**	**Mean±SD in cases**	**Mean±SD in controls**
Vohr et al. 1995 [49]	USA	Prospective cohort	All mothers were screened for GDM in a universal screen program instituted in 1982.	GDM.	Criteria for screening tests for GDM [52]	Treatment with insulin and diet.	Digital measurement.	NR	119; 143	NR	NR
Pribylova et al. 1996 [45]	The Czech Republic	Prospective cohort	Control: offspring of healthy mothers without a family history of diabetes.	T1DM.	Medical diagnosis.	NR	NR	SBP, DBP	148; 31	NR	107.0 ± 14.48 65.7 ± 10.58
Cho et al. 2000 [35]	USA	Prospective cohort	Control: children whose mothers had normal OGTT result.	GDM, preGDM.	Criteria for screening tests for GDM.	Diet controlled unless glycemic targets not achieved, then insulin controlled.	Manual measurement	SBP, DBP	99(GDM: 44, preGDM: 55); 80	118.0 ± 12.0 70.5 ± 9.5	110 ± 11.3 68.9 ± 9.4
Manderson et al. 2002 [41]	UK	Prospective cohort	Control: children ‘without family history of diabetes’.	T1DM.	Medical diagnosis.	Treatment with insulin.	Digital measurement	SBP, DBP	61; 57	103.8 ± 10.2 57.8 ± 6.9	105.8 ± 13.9 58.8 ± 10.5
Bunt et al. 2005 [32]	India	Prospective cohort	Children of a diabetic pregnancy born before the mother developed diabetes.	T2DM.	GWHO.	NR	Manual measurement	SBP, DBP	22; 20	118.0 ± 13.0 63.1 ± 10.2	107.0 ± 10 61.6 ± 8.9
Boney et al. 2005 [31]	Rhode Island-USA	Retrospective cohort	Control: children ‘without family history of diabetes’.	GDM.	Carpenter and Coustan criteria.	Diet controlled unless glycemic target not achieved, then insulin controlled.	Digital measurement	SBP, DBP	81; 77	107.4 ± 10.4 57.9 ± 8.2	104.2 ± 8.6 55.5 ± 7.9
Tam et al. 2008 [47]	HK-CHN	Prospective study	Control: children whose mothers had normal OGTT result.	GDM.	GWHO 1999.	NR	Digital measurement	SBP, DBP	63; 101	91.0 ± 11.7 61.0 ± 5.1	90.0 ± 10.9 58.0±7.1
Buzinaro et al. 2008 [33]	Brazil	Prospective cohort	Control: children whose mothers had normal OGTT result.	GDM.	Diagnosed by OGTT result.	Insulin treatment in 31% of the mothers.	NR	SBP, DBP	23; 27	102.0 ± 13.0 68.0 ± 9.0	101.0 ± 11.0 68.0±10.0
Pirkola et al. 2008 [44]	Oulu-Finland	Prospective cohort	Control: children whose mothers had normal OGTT result.	T1DM, GDM.	National guidelines.	Diet controlled unless glycemic targets not achieved.	NR	SBP, DBP	38(GDM: 22, T1DM: 16); 25	99.2 ± 6.2 60.2 ± 5.4	101.7 ± 7.7 58.1 ±6.6
Wright et al. 2009 [51]	Massachusetts-USA	Prospective cohort	Control: children whose mothers had normal OGTT result.	GDM.	Diagnosed by OGTT result.	Treated with diet, exercise, and in some cases insulin.	Digital measurement	SBP	51; 1035	96.0 ± 11.0 NR	92.0 ± 10.0 NR
Catalano et al. 2009 [34]	USA	Prospective cohort	Control: children whose mothers had normal OGTT result.	GDM.	National Diabetes Data Group criteria.	Diet controlled unless glycemic target not achieved, then insulin controlled.	NR	SBP, DBP	37; 52	110.0 ± 11.0 58.0 ± 7.0	108.0 ± 12.0 60.0 ± 8.0
Krishnaveni et al. 2010 [37]	Mysore-India	Prospective cohort	Control: children whose mothers had normal OGTT result.	GDM.	Carpenter and Coustan criteria.	NR	Digital measurement	SBP, DBP	35; 381	104.54 ± 9.44 59.97 ± 5.8	100.65 ± 8.78 58.25 ± 6.75
Kvehaugen et al. 2010 [39]	Oslo-Norway	Prospective cohort	Cardiovascular health in mother and offspring after pregnancy complications	T1DM, GDM.	GWHO.	NR	Manual measurement	SBP, DBP	22(GDM: 12, T1DM: 10); 17	97.0 ± 7.2 58.6 ± 6.2	98.2 ± 5.7 58.1 ± 4.6
Lindsay et al. 2010 [40]	Scotland	Prospective cohort	FIGS study. All mothers were screened for GDM.	T1DM.	National Guidelines.	NR	Digital measurement	NR	100; 45	NR	NR
West [50] et al. 2011	Colorado-USA	Retrospective cohort	Control: children without intrauterine growth restriction.	T1DM, GDM.	Diagnosed by OGTT result.	NR	NR	SBP, DBP	99(GDM: 91, T1DM: 8); 422	102.9 ± 9.6 69.4 ± 7.7	103.2±10.1 70.1±7.8
Tsadok et al. 2011 [48]	Jerusalem-Israel	Retrospective cohort	All births occurred in western Jerusalem between 1964 and 1976.	GDM.	Medical diagnosis.	NR	Manual measurement	SBP, DBP	293; 59499	121.56 ± 12.30 75.12 ± 7.44	119.84 ± 12.06 73.47±8.30
Rijpert et al. 2011 [46]	Netherlands	Prospective cohort	Investigator blinded to specific characteristics of the pregnancy and neonatal outcome.	T1DM.	Medical diagnosis.	Treatment with insulin.	Digital measurement	SBP, DBP	213; 79	100.4 ± 8.8 58.8 ± 5.8	96.5 ± 8.0 58.1 ± 5.3
Krishnaveni et al. 2015 [38]	India	Prospective cohort	Children whose mothers divided by OGTT results.	GDM.	Medical diagnosis.	NR	Digital measurement	SBP, DBP	26; 165	110.5 ± 8.1 61.7 ± 6.5	109 ± 8.3 61.4 ± 7.0
Tam et al. 2017 [21]	HK-CHN	Prospective cohort	Children whose mothers divided by OGTT results.	GDM.	GWHO 2013.	NR	Digital measurement	SBP, DBP	132; 794	104 ± 8.7 63.0 ± 8.1	102.0 ± 8.9 62.0 ± 7.9
Guttier et al. 2019 [36]	Pelotas- Brazil	Prospective cohort	Data from the hospital interview and the 6- and 11 year follow-ups were used.	GDM.	Medical diagnosis.	NR	Digital measurement	SBP, DBP	Two age groups; 112; 3410 111; 3381	101.53 ± 11.13 62.21 ± 9.34; 115.79 ± 11.34 67.76 ± 8.01	99.07 ± 9.63 60.42 ± 8.75; 113.03 ± 11.25 66.02 ± 8.72
Miranda et al. 2019 [42]	Porto- Portugal	Prospective cohort	Recruited 8647 children from all public maternity units of Porto, Portugal, during 2005 to 2006.	T1DM, T2DM, and GDM.	Medical diagnosis.	NR	Manual measurement	SBP, DBP	Three age group: 298; 4223 392; 5178 365; 4760	98.60 ± 8.49 58.01 ± 8.18; 106.16 ± 9.18 70.50 ± 7.33; 111.30 ± 9.77 70.22 ± 7.16	98.25 ± 8.37 57.73 ± 8.10; 105.47 ± 8.84 69.99 ± 7.58; 109.59 ± 9.31 69.26 ± 6.91
Lu et al. 2019 [18]	Tianjin- CHN	Prospective cohort	An urban universal screening of GDM.	GDM.	GWHO 1999.	Treatment with insulin.	Manual measurement	SBP, DBP	578; 578	97.2 ± 8.9 60.3 ± 8.2	94.3 ± 8.3 59.9 ± 6.6
Perng et al. 2020 [43]	Colorado- USA	Prospective cohort	/	GDM.	Medical diagnosis.	diet and/or exercise only, diet and/or exercise with insulin, and insulin only.	Digital measurement	SBP	92; 505	111.38 ± 8.74 NR	108.98 ± 11.21 NR

*NR* not reported, *OGTT* oral glucose tolerance test, *GWHO* guidelines of WHO, *ACOG* The American College of Obstetricians and Gynecologists, *GDM* gestational diabetes mellitus, *T1DM* Type 1 diabetes mellitus, *T2DM* type 2 diabetes mellitus, *SBP* systolic blood pressure, *DBP* diastolic blood pressure

### Methodological quality and risk of bias for the included studies

In addition to the uneven distribution of studies across socioeconomic contexts and regions, the use of varying methodological approaches also may have contributed to the risk of bias. For example, studies reporting the “exposure assess before to outcome measurement”, “different levels of the exposure of interest”, “repeated exposure assessment”, and “ blinding of outcome assessors” were considered potentially to have a high risk of bias. Five (21.7%) studies [18, 21, 38, 39, 46] reported blinding of outcome assessment. 10 (43.5%) studies [18, 31, 32, 42, 43, 45, 48–51] reported the exposure assessed prior to outcome measurement. Eight (34.8%) studies [21, 36, 38, 40, 42, 45, 46, 51] reported different levels of the exposure of interest and 12 (52.2%) studies [18, 21, 31, 35, 36, 38, 40, 42, 43, 45–47] reported the repeated exposure assessment. The quality assessment of included studies is shown in Table S2, Additional file 1.

### Offspring blood pressure

A summary of the meta-analysis results from the included studies is presented in figures below. In these diagrams, results regarding the offspring BP of the mothers with HIP are presented. Furthermore, where the adequate data was available in primary studies, the diagnosed subtypes of HIP were analyzed respectively.

#### Offspring of mothers with HIP

**Fig. 2:**
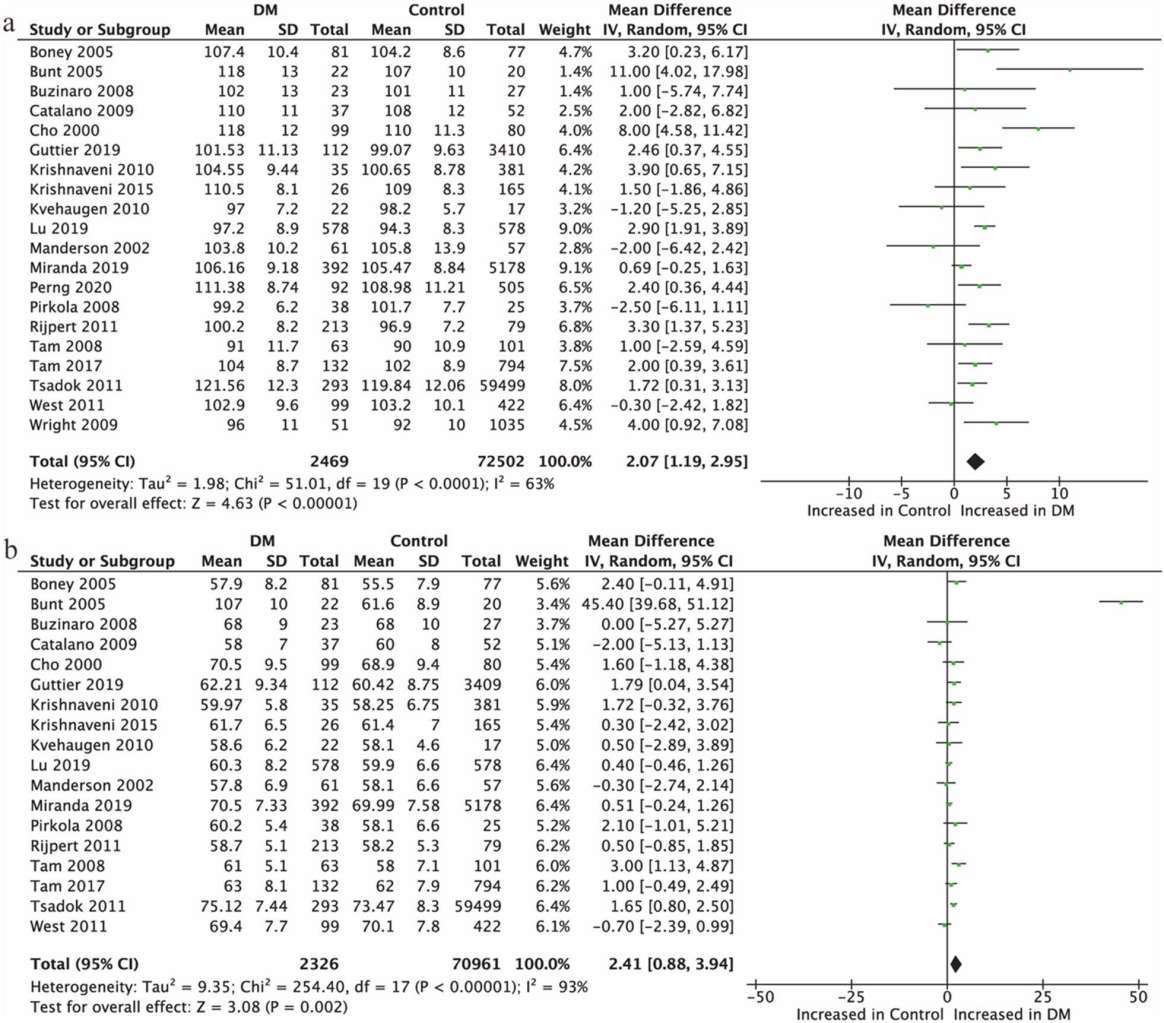
Forest plot showing the association between HIP and offspring BP. **a** SBP; **b** DBP. IV, inverse variance

Overall, 20 studies [18, 21, 31–39, 41–44, 46–48, 50, 51] provided data on SBP in offspring of mothers with HIP, and 18 [18, 21, 31–39, 41, 42, 44, 46–48, 50] on DBP. It can be seen from forest diagrams that both SBP and DBP were significantly higher in offspring of mothers with HIP than those in their controls (SBP: 2.07 mmHg, 95% CI [1.19, 2.95], P < 0.001; Fig. 2a. DBP: 2.41 mmHg, 95% CI [0.88, 3.94], P < 0.001; Fig. 2b).

#### Offspring of T1DM

**Fig. 3:**
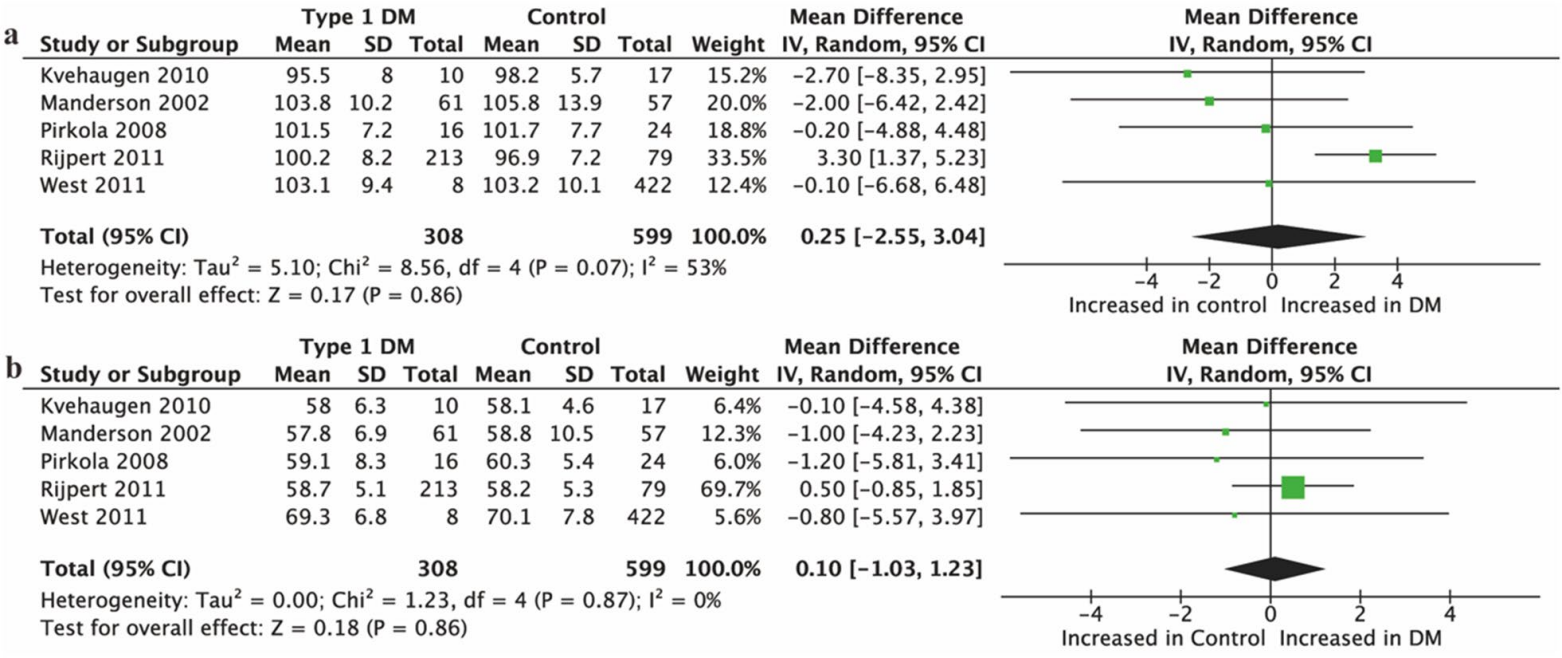
Forest plot showing the association between T1DM and offspring BP. **a** SBP; **b** DBP. IV, inverse variance

In total, five studies [39, 41, 44, 46, 50] reported data on BP in children born to T1DM. There was no difference in either SBP or DBP between offspring of women with T1DM and controls (SBP: 0.25 mmHg, 95% CI [− 2.55, 3.04], P = 0.86; Fig. 3a. DBP: 0.10 mmHg, 95% CI [− 1.03, 1.23], P = 0.86; Fig. 3b).

#### Offspring of women with GDM

**Fig. 4:**
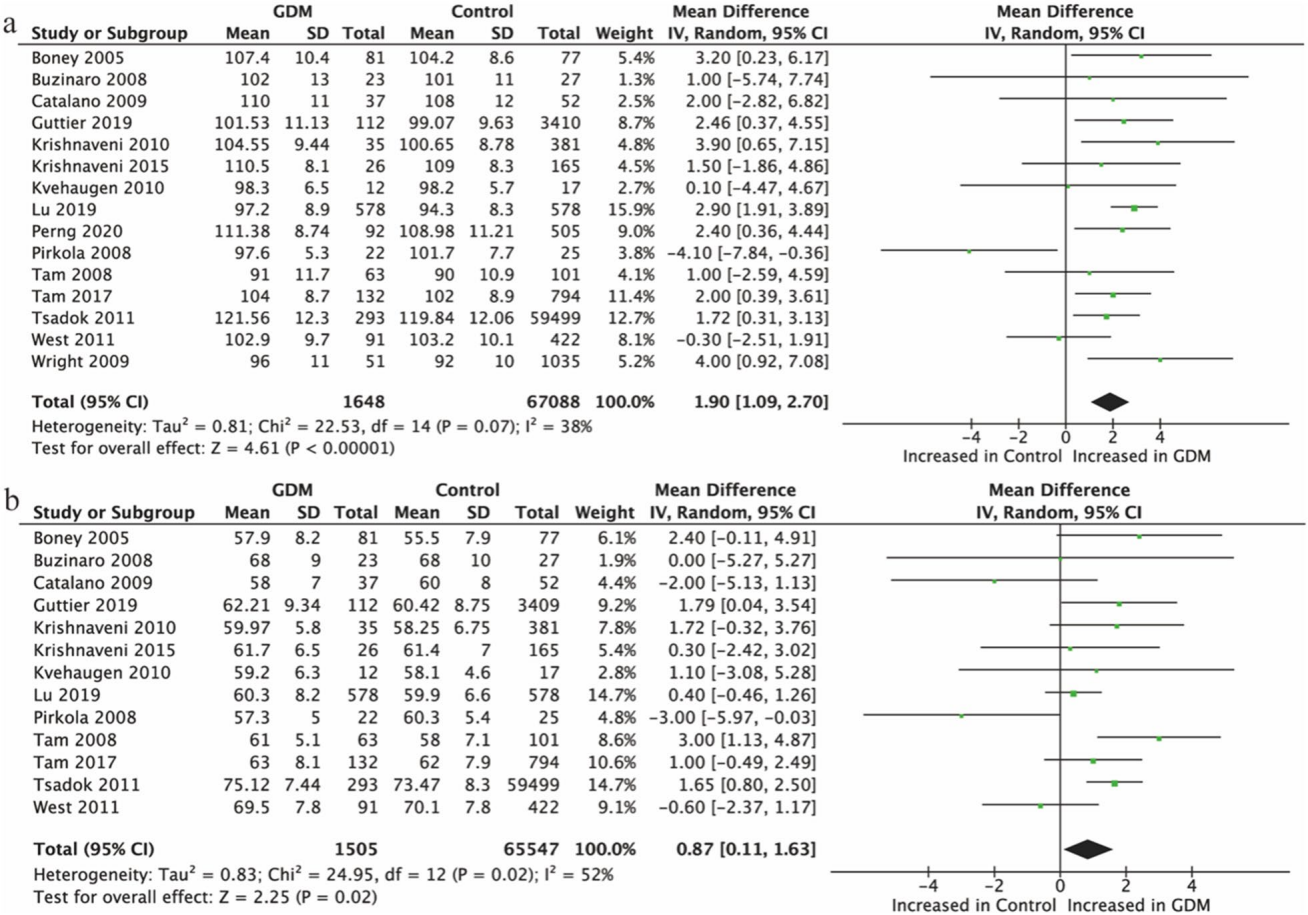
Forest plot showing the association between GDM and offspring BP. **a** SBP; **b** DBP. IV, inverse variance

There were 15 studies [18, 21, 31, 33, 34, 36–39, 43, 44, 47, 48, 50, 51] that reported data on SBP and 13 [18, 21, 31, 33, 34, 36–39, 44, 47, 48, 50] on DBP under the exposure of maternal gestational diabetes. Both SBP and DBP in Offspring of women with GDM were higher than those in controls (SBP: 1.90 mmHg, 95% Cl [1.09, 2.70], P < 0.001; Fig. 4a. DBP: 0.87 mmHg, 95% Cl [0.11, 1.63], P = 0.02; Fig. 4b).

### Subgroup analysis

Subgroup analyses of offspring of mothers with HIP were performed according to sex, the WHO region, economic level, published year, insulin treatment, and BP measurement. The detailed analyzed data for the offspring BP subgroups are listed in Table 2.

**Table 2:** Subgroups analysis of BP in offspring of women with HIP

BP outcomes	No. of studies	No. of experimental group	No. of control group	Effect size (mmHg)	P-value	Heterogeneity^*^
SBP						
Sex						
Male	7	382	39201	2.12 [0.43, 3.81]	0.01	P = 0.11; I^2^ = 42%
Female	7	328	24825	2.99 [1.59, 4.38]	< 0.001	P = 0.36; I^2^ = 9%
WHO region						
EUR	2	34	42	− 2.21 [− 6.31, 1.88]	0.29	P = 0.16; I^2^ = 49%
AMR	7	487	5528	2.08 [0.97, 3.19]	< 0.001	P = 0.34; I^2^ = 12%
SEAR	2	61	546	2.74 [0.39, 5.09]	0.02	P = 0.31; I^2^ = 1%
WPR	3	773	1473	2.57 [1.74, 3.39]	< 0.001	P = 0.44; I^2^ = 0%
EMR	1	293	59499	/	/	/
WB region						
HICs	10	874	62527	1.44 [0.32, 2.55]	0.01	P = 0.06; I^2^ = 46%
UMICs	3	713	4015	2.79 [1.90, 3.68]	< 0.001	P = 0.81; I^2^ = 0%
LMICs	2	61	546	2.74 [0.39, 5.09]	0.02	P = 0.31; I^2^ = 1%
Published year						
Before 2010	8	324	1715	1.58 [-0.40, 3.56]	0.12	P = 0.04; I^2^ = 53%
After 2011	7	1324	65373	2.07 [1.35, 2.80]	< 0.001	P = 0.27; I^2^ = 21%
Insulin treatment						
Insulin-treated	7	884	2299	2.04 [0.44, 3.65]	0.01	P = 0.03; I^2^ = 57%
Insulin-untreated	8	764	64788	1.35 [0.65, 2.06]	< 0.001	P = 0.23; I^2^ = 24%
BP measurement						
Digital measurement	8	592	6468	2.45 [1.59, 3.32]	< 0.001	P = 0.86; I^2^ = 0%
Manual measurement	3	883	60094	2.30 [1.21, 3.39]	< 0.001	P = 0.24; I^2^ = 29%
DBP						
Sex						
Male	6	333	38953	1.76 [0.89, 2.63]	< 0.001	P = 0.61; I^2^ = 0%
Female	6	285	24567	1.71 [0.44, 2.99]	0.008	P = 0.16; I^2^ = 36%
WHO region						
EUR	2	34	42	− 1.22 [− 5.21, 2.76]	0.55	P = 0.12; I^2^ = 59%
AMR	5	344	3987	0.51 [− 1.10, 2.11]	0.54	P = 0.08; I^2^ = 51%
SEAR	2	61	546	1.21 [− 0.42, 2.84]	0.15	P = 0.41; I^2^ = 0%
WPR	3	773	1473	1.27 [− 0.12, 2.66]	0.07	P = 0.05; I^2^ = 68%
EMR	1	293	59499	/	/	/
WB region						
HICs	8	731	60987	0.70 [− 0.52, 1.91]	0.26	P = 0.003; I^2^ = 67%
UMICs	3	172	3488	0.21 [− 2.45, 2.86]	0.88	P = 0.11; I^2^ = 54%
LMICs	2	61	546	1.21 [− 0.42, 2.84]	0.15	P = 0.41; I^2^ = 0%
Published year						
Before 2010	7	273	680	0.68 [− 1.10, 2.47]	0.45	P = 0.01; I^2^ = 64%
After 2011	6	1232	64967	0.88 [0.17, 1.59]	0.02	P = 0.14; I^2^ = 39%
Insulin treatment						
Insulin-treated	6	943	854	0.03 [− 1.26, 1.32]	0.97	P = 0.09; I^2^ = 48%
Insulin-untreated	6	532	1694	1.74 [0.45, 3.03]	0.008	P = 0.01; I^2^ = 68%
BP measurement						
Digital measurement	6	449	4927	1.71 [0.92, 2.50]	< 0.001	P = 0.54; I^2^ = 0%
Manual measurement	3	883	60094	1.03 [0.01, 2.05]	0.05	P = 0.13; I^2^ = 51%

All effect sizes’were calculated by subgroups’ mean difference (IV, Random, 95% CI)

*ODM* Offspring of women with diabetes mellitus, *OGDM* Offspring of women with gestational diabetes mellitus, *EUR* European Region, *AMR* Region of the Americas, *SEAR* South-East Asia Region, *WPR* Western Pacific Region, *EMR* Eastern Mediterranean Region

^*^P value from the χ2 test

SBP and DBP of offspring of women with HIP in both males and females were higher than those in control groups (Male SBP: 2.12 mmHg, 95% CI [0.43, 3.81]; P = 0.01; Male DBP: 1.76 mmHg, 95% CI [0.89, 2.63]; P < 0.001. Female SBP: 2.99 mmHg, 95% CI [1.59, 4.38]; P < 0.001; Female DBP: 1.71 mmHg, 95% CI [0.44, 2.99], P = 0.008).

With regard to the subgroup analyses of offspring of mothers with HIP based on the WHO region, SBP of Offspring of women with gestational diabetes mellitus (OGDM) among AMR, SEAR, and WPR were all significantly higher than that of their counterparts in other regions (AMR: 2.08 mmHg, 95% CI [0.97, 3.19], P < 0.001; SEAR: 2.74 mmHg, 95% CI [0.39, 5.09], P = 0.02; WPR: 2.57 mmHg, 95% CI [1.74, 3.39], P < 0.001), while such difference did not exist between SBP of cases and controls in EUR (− 2.21 mmHg, 95% CI [− 6.31, 1.88]; P = 0.29). As for DBP, no difference was found between offspring of women with HIP and their controls in all EUR, AMR, SEAR, and WPR (EUR: − 1.22 mmHg, 95% CI [− 5.21, 2.76], P = 0.55; AMR: 0.51 mmHg, 95% CI [− 1.10, 2.11], P = 0.54; SEAR: 1.21 mmHg, I [− 0.42, 2.84], P = 0.15; WPR: 1.27 mmHg, 95% CI [− 0.12, 2.66], P = 0.07).

In the subgroup analyses of offspring of women with HIP based on income, this study found that SBP was higher than that in controls in HICs, UMICs, and LMICs. (HICs: 1.44 mmHg 95% CI [0.32, 2.55]; P = 0.01; UMICs: 2.79 mmHg, 95% CI [1.90, 3.68], P < 0.001; LMICs: 2.74 mmHg, 95% CI [0.39, 5.09]; P = 0.02). However, there was no significant difference between DBP of offspring of women with HIP and that in controls in all HICs, UMICs, LMICs (HICs: 0.70 mmHg 95%CI [− 0.52, 1.91]; P = 0.26. UMICs: 0.21 mmHg, 95% CI [− 2.45, 2.86]; P = 0.88; LMICs: 1.21 mmHg, 95% CI [− 0.42, 2.84]; P = 0.15).

For the analyses based on published year, our study found that only estimated BP in maternal HIP group published after 2011 was significantly higher than that in controls (SBP: 2.07 mmHg, 95% CI 1.35, 2.80], P < 0.001; DBP: 0.88 mmHg, 95% CI [0.17, 1.59], P = 0.02) whereas results showed no difference of BP in studies before 2010 (SBP: 1.58 mmHg, 95% CI [− 0.40, 3.56], P = 0.12. DBP: 0.68 mmHg, 95% CI [− 1.10, 2.47], P = 0.97).

While grouping by insulin treatment status, SBP in insulin-treated group and BP in insulin-untreated group were both higher than those in controls (SBP insulin-treated: 2.04 mmHg, 95% CI [0.44, 3.65], P = 0.01; SBP insulin-untreated: 1.35 mmHg, 95% CI [0.65, 2.06], P < 0.001; DBP insulin-untreated: 1.74 mmHg, 95% CI [0.45, 3.03], P = 0.008). By contrast, there was no significant difference between DBP in the insulin-treated group and that in controls (0.03 mmHg, 95% CI [− 1.26, 1.32], P = 0.97).

For the subgroup analysis of offspring of women with HIP based on BP measurement, BP assessed by digital and manual BP measurement was both higher than in controls (SBP Digital measurement: 2.45 mmHg, 95% CI [1.59, 3.32], P < 0.001; SBP manual measurement: 2.30 mmHg, 95% CI [1.21, 3.39], P < 0.001; DBP digital measurement: 1.71 mmHg, 95% CI [0.92, 2.50], P < 0.001; DBP manual measurement: 1.03 mmHg, 95% CI [0.01, 2.05], P = 0.05).

### Publication bias of the included studies

Funnel plots were visually inspected to assess the potential publication bias.

**Fig. 5:**
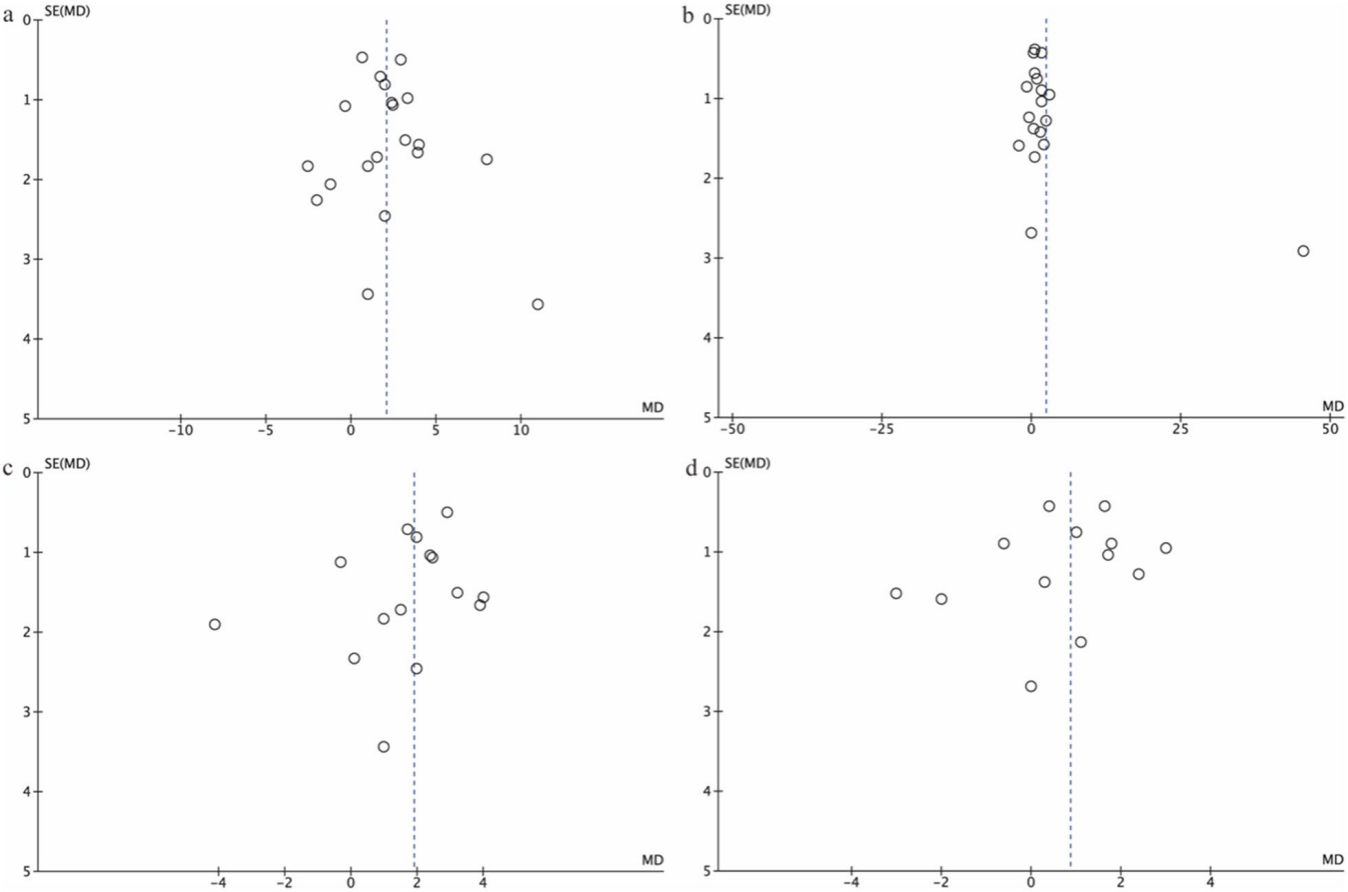
BP funnel plot **a** HIP SBP; **b** HIP DBP; **c** OGDM SBP; **d** OGDM DBP

It is observable that the distribution of studies was generally consistent across funnel plots of maternal HIP and GDM as outcomes. Visually, all four funnel plots had an overall symmetrical presentation, representing little effect from publication bias. We could not use a funnel plot to assess the bias because fewer than 10 studies of offspring of mothers with T1DM were reported (Fig. 5).

## Discussion

This systematic review and meta-analysis aimed to assess the potential risk of hypertension in the offspring of HIP. In general, there was an increased danger of higher DBP and SBP in offspring born to mothers with general HIP and GDM.

Compared with the article of Aceti et al. we have synthesized current studies in more comprehensive ways. Up-to-date evidence was added to the previous ten studies [31, 33, 34, 37, 39, 44, 47, 48, 50, 51] included in the meta-analysis of Aceti et al. we have the rest three studies [35, 41, 46] because they mismatched our inclusion criteria [53].

The results of our study reflect the recent global health status considering the ongoing social changes and research advances better. Furthermore, our research has broadly explored the subgroup analyses which suggested that the linkage between offspring BP and maternal HIP may vary depending on the sex of offspring, BP measurement, insulin treatment status, income, region, and the published year of articles.

More specifically, SBP and DBP appeared to have more substantial and persistent effects on female offspring. Despite the protective effect of endogenous estrogen on BP regulation [54], recent data has cast doubt on the actual protective function for females. We recently found that women have higher BP than men among individuals born prematurely [55]. In line with this evidence, intracardiac studies have also shown that young women with characteristics associated with metabolic syndrome have the highest risk of acute myocardial infarction [56]. It may be because the major estrogen distribution changes throughout puberty together with the menstrual cycle may also affect BP [57]. Therefore, based on mixed pictures of empirical studies, the results of gender differences here should also be carefully used.

As for economic status, it is commonly believed that higher income or socio-economic status might lead to a healthier lifestyle. Aligning with this common sense, mothers with higher socio-economic status have been demonstrated to be at a lower risk of GDM [58]. However, in our research, there were no varied patterns of influence from GDM on offspring DBP and SBP across HICs, UMICs, and LMICs. For one, it could be explained that many low-income countries are currently experiencing demographic and epidemiological transitions as well as lifestyle changes.

Related to this, the BP seemed to be consistently influenced by maternal GDM across regions. Our study defined the region of mothers by their ethnicity, that is, the maternal region of birth. Therefore, the possible biological variances among women from various ethnicities were allowed for inspection in our research. In contrast to our result, previous research suggested maternal diabetes affected child health outcomes differently in Australia and Caucasians [59]. We suspect that there are two reasons for such inconsistency. One is that there are only small pieces of literature included in the regional subgroup [39, 44], and the other lies in the few samples in the experimental and control group in the literature. Therefore, future research still warrants uncovering this mechanism [58].

An interesting phenomenon from our result is that the effect of HIP on offspring’s DBP varied by maternal use of insulin medication. However, another study found no meaningful differences in long-term childhood growth among the offspring of women with GDM treated with insulin compared to nutritional therapy groups [58]. We were constrained from yielding a definitive conclusion due to the unspecified BP measures [60, 61] in several studies and the limitation of sample size. These effects can also explain our finding: there was no significant difference in DBP in the pre-2010 studies, whereas post-2011 studies did. While looking into post-2011 studies, there were larger sample sizes, and mix-methods of BP measurements were more frequently used, including manual sphygmomanometers and automated devices [36, 62]. Furthermore, according to the quality assessment, the methods were of potential risks of bias. As a result, when the analysis was limited to high-quality studies with minimal heterogeneity [63], treatment status and published no longer influenced the relationship between maternal HIP and offspring BP. Therefore, more examinations with high quality should be carried out in the future to distinguish confounds from real effects.

## Strengths and limitations

Our study was more comprehensive than previous studies. Compared with the meta-analysis published by Aceti et al. in 2012, which elucidated the association between maternal diabetes and offspring BP [22], we have updated the original article based on existing eligible research. In addition, more meaningful subgroup analyses of OGDM were perform according to the WHO region, economic level, published year, insulin treatment, and BP measurement.

The present study had several limitations. Firstly, owing to a lack of previous data on T1DM, most of the above conclusions are of limited applicability for those mother-child dyads until more empirical studies are conducted. Secondly, the lifestyle variables, such as diet, physical activities, and sleep patterns were lacking in original studies. Therefore, the behavioral factors during the generational transmission of such adverse health conditions remain unclear. Lastly, we failed to demonstrate the age distribution of children in relation to the BP outcomes, which may have been able to trace the dynamic developmental trajectory of offspring in the meta-analysis.

Several implications of this review are pointed out for future research and practice. Firstly, the quality of assessment suggested superior quality may be achieved through adherence to sufficient measurement reports, multiple levels of HIP estimation, the blindness of assessors, etc. Additionally, based on the close relationship between offspring BP and maternal HIP, its pathology process is needed for the next-step research. Besides, the epigenetic pathways may contribute to our understanding of the underlying reasons by introducing more psycho-social or genetic factors into this research scope. In this way, those studies may provide new insights into the pathogeneses of human diseases and tailored prevention by following the concept of Developmental Origins of Health and Disease (DOHaD). Nevertheless, the T1DM condition did not relate to the higher BP of offspring. Although the study's amount of T1DM is relatively small, it may reveal the indirect effect of maternal insulin resistance on the offspring hypertension, a vascular complication of T1DM, is limited [64]. This finding has valuably provided evidence for the “fetal programming” hypothesis of maternal diabetes on the development of metabolic disease [22, 36, 65].

## Conclusion

In summary, our review indicates that GDM may result in elevated systolic and diastolic BP in the offspring, providing evidence for fetal cardiovascular risks brought by HIP. Moreover, our study revealed that BP is more seriously impacted after 2011 or when mothers are insulin-untreated. All these factors imply that changes in epigenetic mechanisms may influence the initiation and progression of metabolic diseases that warrant future research. Crucially, we also stress the importance of medical treatment and health promotion adapted to social development.

## Supplementary Information

**Additional file 1.** **Text S1** Comprehensive search strategy. **Table S1** The process of literature review and data extraction. **Table S2** The quality assessment of included studies.

## Data Availability

The datasets used and/or analyzed during the current study are available from the corresponding author on reasonable request.

## Abbreviations

HIP: Hyperglycemia in pregnancy
BP: Blood pressure
CI: Confidence interval
SBP: Systolic blood pressure
MD: Mean difference
DBP: Diastolic blood pressure
GDM: Gestational diabetes mellitus
DIP: Diabetes mellitus in pregnancy
T1DM: Type 1 diabetes mellitus
T2DM: Type 2 diabete mellitus
PRISMA: Preferred reporting items for systematic reviews and meta-analyses
MOOSE: Meta-analyses of observational studies in epidemiology
WHO: World Health Organization
WB: World bank
RevMan: Review manager
EUR: European region
EMR: Eastern mediterranean region
AMR: Region of the americas
SEAR: South-East Asia region
WPR: Western Pacific region
HICs: High-income countries
UMICs: Upper-middle-income countries
LMICs: Lower-middle-income countries
OGDM: Offspring of women with gestational diabetes mellitu
DOHaD: Developmental origins of health and disease
