# Supplementary material for: Hyperglycaemia in pregnancy and offspring blood pressure: a systematic review and meta-analysis
Source: Diabetol Metab Syndr. 2023 Jan 19;15:10. doi: 10.1186/s13098-023-00978-2 (PMC9850544; doi:10.1186/s13098-023-00978-2)
Supplement: Supplementary file 1 — Additional file 1. Text S1 Comprehensive search strategy. Table S1 The process of literature review and data extraction. Table S2 The quality assessment of included studies. [file 13098_2023_978_MOESM1_ESM.docx]

**Additional file**

**Hyperglycaemia in pregnancy and offspring blood pressure: a systematic review and meta-analysis**

Additional file **Text S1**. GD comprehensive search strategy.

Additional file **Table S1.** The process of literature review and data extraction.

Additional file **Table S2.** The quality assessment of included studies.

This supplemental material has been provided by the authors to give readers additional information about their work.

**Additional file Text S1.** GD comprehensive search strategy.

PubMed, MEDLINE (1950-), EMBASE (1980-)

gestational diabetes: gestational diabetes OR GDM OR pregnancy glycaemic index OR Pregnancy-Induced Diabetes

Maternal DM: (maternal OR pregnan* or gestation* or prenatal) AND (diabetes OR diabetic)

BP: blood pressure OR hypertension

Children: child* OR adolescen* OR offspring*

Not: twins

Limit to: humans

PubMed

Access Date: 09 Feb 2021

Search Terms:

(gestational diabetes[Title/Abstract] OR GDM[Title/Abstract] OR pregnancy glycaemic index[Title/Abstract] OR Pregnancy Induced Diabetes[Title/Abstract]) AND (blood pressure[Title/Abstract] OR hypertension[Title/Abstract]) AND (child*[Title/Abstract] OR adolescen*[Title/Abstract] OR offspring*[Title/Abstract]) NOT twin*[Title/Abstract]

(maternal[Title/Abstract] OR pregnan*[Title/Abstract] OR gestation*[Title/Abstract] OR prenatal[Title/Abstract]) AND (diabetes[Title/Abstract] OR diabetic[Title/Abstract]) AND (blood pressure[Title/Abstract] OR hypertension[Title/Abstract]) AND (child*[Title/Abstract] OR adolescen*[Title/Abstract] OR offspring*[Title/Abstract]) NOT twin*[Title/Abstract]

Total: 369

Medline

Access Date: 09 Feb 2021

# ▲ Searches Results

1 (maternal or pregnan* or gestation* or prenatal).ab,ti. 791406

2 (diabetes or diabetic).ab,ti. 645795

3 exp Diabetes Mellitus, Type 1/ or exp Diabetes, Gestational/ or exp Diabetes Mellitus/ or exp Diabetes Mellitus, Type 2/ 436304

4 (blood pressure or hypertension).ab,ti. 595679

5 Blood Pressure/ 277500

6 (child* or adolescen* or offspring*).ab,ti. 1653837

7 2 or 3 714394

8 4 or 5 717588

9 1 and 6 and 7 and 8 1444

10 limit 9 to (english language and humans and journal article and medline) 998

Embase

Access Date: 09 Feb 2021

Maternal DM: (maternal OR pregnan* or gestation* or prenatal) AND (diabetes OR diabetic)

BP: (blood pressure OR hypertension)

Children: (child* OR adolescen* OR offspring*)

Total: 1316

GDM and HP Inclusion and exclusion criteria

Inclusion criteria

(1) Observational population-based study: cross-sectional, case-control, or cohort;

(2) The exposure of interest should be diabetes during pregnancy (type 1, type 2 and gestational diabetes);

(3) The outcome of interest should be blood pressure or hypertension in offspring/children/adolescents;

(4) For childhood hypertension, odds ratio (OR) or relative risk (RR) with corresponding 95% confidence interval (CI) or standard err (SE) should have been provided; For blood pressure, n, mean and sd should have been provided

Exclusion criteria

(1) In vitro studies, animal studies, randomized controlled trials, and non-original studies (reviews, news and commentaries)

(2) Multiple publications of the same study (the one with the most recent or most comprehensive results should be kept).

**Additional file Table S1.** The process of literature review and data extraction.

|  | Step 1: Conduct the database searches and review titles | | | | Step 2: Review abstracts | | Step 3: Review full text | | | | | | |
| --- | --- | --- | --- | --- | --- | --- | --- | --- | --- | --- | --- | --- | --- |
|  |  | 1.1.  Search terms | 1.2.  Please download all relevant titles into a separate Notes Express file for each database search. Email a copy. | 1.3.  Merge the 3 files into one, get rid of all duplicate titles. | 2.1.  Go through the titles and abstracts of all the relevant titles, and apply the exclusion criteria below. Note the number of papers that were excluded for each for the 6 reasons. Choose the most relevant reason for each paper. | 2.2.  Report the total number of abstracts retained | 3.1. Download the full text file for all retained abstracts. | 3.2.  Go through the files. Apply the exclusion criteria below. Note the number of papers that were excluded for each fo the 6 reasons. Choose the most relevant reason for each paper. | | | | | 3.3. Report the total number of full text articles retained |
|  |  | Titles | | | Abstracts | Abstracts retained | Full text | | | | | | Full text retained |
| DATABASES |  |  | No. of titles | No. of titles overall | Reasons for discarding papers at the abstract level |  | Insufficient information on methods and results | Reasons for discarding papers at the full text level | | | | |  |
|  | Access date |  |  |  | A |  | 0 | 1 | 2 | 3 | 4 | 5 |  |
| PubMed | 9-Feb-21 | see word file | 1071 | 2362 | 2312 | 50 | 5 | 4 | 1 | 3 | 8 | 6 | 23 |
| MEDLINE (1950-) | 9-Feb-21 | see word file | 998 |  |  |  |  |  |  |  |  |  |  |
| EMBASE (1980-) | 9-Feb-21 | see word file | 1316 |  |  |  |  |  |  |  |  |  |  |

| **REASONS FOR EXCLUSION at the ABSTRACT LEVEL** |
| --- |

A: Studies that were not observational, population-based, whose exposure was not maternal diabetes, or outcome was not blood pressure in children

**REASONS FOR EXCLUSION at the FULL-TEXT LEVEL**

0: Papers with no full-text

1: Studies that were not observational or population-based

2: Studies where exposure was not maternal diabetes

3: Studies where outcome was not blood pressure in children

4: Papers where the relevant outcome data were not available

5: Multiple publications of the same investigation

**Additional file Table S2.** The quality assessment of included studies.

| ID | Author | Published Year | Items | | | | | | | | | | | | | | Sum |
| --- | --- | --- | --- | --- | --- | --- | --- | --- | --- | --- | --- | --- | --- | --- | --- | --- | --- |
|  |  |  | 1. | 2. | 3. | 4. | 5. | 6. | 7. | 8. | 9. | 10. | 11. | 12. | 13. | 14. |  |
| 1 | Vohr et al. | 1995 | 1 | 1 | 1 | 1 | 1 | 1 | 1 | 0 | 1 | NR | 1 | NR | 1 | 1 | 11 |
| 2 | Pribylova et al. | 1996 | 1 | 1 | 1 | 1 | 1 | 1 | 1 | 1 | 1 | 1 | 1 | NR | 1 | 1 | 13 |
| 3 | Cho et al. | 2000 | 1 | 1 | 1 | 1 | 1 | 0 | 1 | 0 | 1 | 1 | 1 | NR | 1 | 1 | 11 |
| 4 | Manderson et al. | 2002 | 1 | 1 | 1 | 1 | 1 | 0 | 1 | 0 | 1 | 0 | 1 | NR | 1 | 1 | 10 |
| 5 | Bunt et al. | 2005 | 1 | 1 | 1 | 1 | NR | 1 | CD | 0 | 1 | NR | 1 | NR | 1 | 1 | 9 |
| 6 | Boney et al. | 2005 | 1 | 1 | 1 | 1 | 1 | 1 | 1 | 0 | 1 | 1 | 1 | NR | 1 | 1 | 12 |
| 7 | Tam et al. | 2008 | 1 | 1 | 1 | 1 | 1 | 0 | 1 | 0 | 1 | 1 | 1 | NR | 1 | 1 | 11 |
| 8 | Buzinaro et al. | 2008 | 1 | 1 | 1 | 1 | 0 | 0 | 1 | 0 | 1 | NR | 1 | NR | 1 | 1 | 9 |
| 9 | Pirkola et al. | 2008 | 1 | 1 | 1 | 1 | 0 | 0 | 1 | 0 | 1 | 0 | 1 | NR | 1 | 1 | 9 |
| 10 | Wright et al. | 2009 | 1 | 1 | 1 | 1 | 1 | 1 | 1 | 1 | 1 | 0 | 1 | 0 | 1 | 1 | 12 |
| 11 | Catalano et al. | 2009 | 1 | 1 | 1 | 1 | 0 | 0 | 1 | 0 | 1 | 0 | 1 | NR | 1 | 1 | 9 |
| 12 | Krishnaveni et al. | 2010 | 1 | 1 | 1 | 1 | 1 | 0 | 1 | 0 | 1 | 0 | 1 | NR | 1 | 1 | 10 |
| 13 | Kvehaugen et al. | 2010 | 1 | 1 | 1 | 1 | 0 | 0 | 1 | 0 | 1 | 0 | 1 | 1 | 1 | 1 | 10 |
| 14 | Lindsay et al. | 2010 | 1 | 1 | 1 | 1 | 0 | 0 | 1 | 1 | 1 | 1 | 1 | NR | 1 | 1 | 11 |
| 15 | West et al. | 2011 | 1 | 1 | 1 | 1 | 0 | 1 | CD | 0 | 1 | 0 | 1 | 0 | 1 | 1 | 9 |
| 16 | Tsadok et al. | 2011 | 1 | 1 | 1 | 1 | 1 | 1 | 1 | 0 | 1 | 0 | 1 | 0 | 1 | 1 | 11 |
| 17 | Rijpert et al. | 2011 | 1 | 1 | 1 | 1 | 1 | 0 | 1 | 1 | 1 | 1 | 1 | 1 | 1 | 1 | 13 |
| 18 | Krishnaveni et al. | 2015 | 1 | 1 | 1 | 1 | NR | 0 | 1 | 1 | 1 | 1 | NR | 1 | 1 | 1 | 11 |
| 19 | Tam et al. | 2017 | 1 | 1 | 1 | 1 | NR | 0 | 1 | 1 | 1 | 1 | 1 | 1 | 1 | 1 | 12 |
| 20 | Guttier et al. | 2019 | 1 | 1 | 1 | 1 | 1 | 0 | 1 | 1 | 1 | 1 | 1 | NR | 1 | 1 | 12 |
| 21 | Miranda et al. | 2019 | 1 | 1 | 1 | 1 | 1 | 1 | 1 | 1 | 1 | 1 | 1 | NR | 1 | 1 | 13 |
| 22 | Lu, J et al. | 2019 | 1 | 1 | 1 | 1 | 1 | 1 | 1 | 0 | 1 | 1 | 1 | 1 | 1 | 1 | 13 |
| 23 | Perng et al. | 2020 | 1 | 1 | 1 | 1 | NR | 1 | 1 | 0 | 1 | 1 | 1 | NR | 1 | 1 | 11 |

YES=1; NO=0; CD, cannot determine; NA, not applicable; NR, not reported. <https://www.nhlbi.nih.gov/health-topics/study-quality-assessment-tools>

1. Was the research question or objective in this paper clearly stated?

2. Was the study population clearly specified and defined?

3. Was the participation rate of eligible persons at least 50%?

4. Were all the subjects selected or recruited from the same or similar populations (including the same time period)? Were inclusion and exclusion criteria for being in the study prespecified and applied uniformly to all participants?

5. Was a sample size justification, power description, or variance and effect estimates provided?

6. For the analyses in this paper, were the exposure(s) of interest measured prior to the outcome(s) being measured?

7. Was the timeframe sufficient so that one could reasonably expect to see an association between exposure and outcome if it existed?

8. For exposures that can vary in amount or level, did the study examine different levels of the exposure as related to the outcome (e.g., categories of exposure, or exposure measured as continuous variable)?

9. Were the exposure measures (independent variables) clearly defined, valid, reliable, and implemented consistently across all study participants?

10. Was the exposure(s) assessed more than once over time?

11. Were the outcome measures (dependent variables) clearly defined, valid, reliable, and implemented consistently across all study participants?

12. Were the outcome assessors blinded to the exposure status of participants?

13. Was loss to follow-up after baseline 20% or less?

14. Were key potential confounding variables measured and adjusted statistically for their impact on the relationship between exposure(s) and outcome(s)?
